# Paediatric massage for treatment of acute diarrhoea in children: a meta-analysis

**DOI:** 10.1186/s12906-018-2324-4

**Published:** 2018-09-18

**Authors:** Li Gao, Chunhua Jia, Huiwen Huang

**Affiliations:** Beijing University of Chinese Medicine, No. 11 North 3rd Ring East Road, Chaoyang District, Beijing, 100029 China

**Keywords:** Massage therapy, Children, Acute diarrhoea, Meta-analysis

## Abstract

**Background:**

Massage therapy has been used by many traditional Chinese medicine physicians to treat acute diarrhoea in children. Since no relevant systematic reviews assessed the clinical effectiveness or the risk of massage therapy, in this study, a meta-analysis was conducted to evaluate the efficacy of paediatric massage for the treatment of acute diarrhoea in children.

**Methods:**

In this meta-analysis, paediatric patients who were diagnosed with acute diarrhoea were included. Interventions using massage therapy alone or combined with other non-pharmacological approaches were included, while in the control groups, patients received pharmacotherapy. The primary outcome was clinical effective rate. Seven databases were used in our research, and the following search terms were used: (massage OR tui na OR manipulation OR acupressure) AND (infant OR child OR baby OR paediatrics) AND (diarrhoea OR diarrhoea) AND (randomized controlled trial). The search date was up to April 30, 2018.

**Results:**

A total of 26 studies encompassing 2644 patients were included in this meta-analysis. It was shown that paediatric massage was significantly better than pharmacotherapy in treating acute diarrhoea in children in terms of clinical effective rate (*n* = 2213, RR = 1.20, 95% CI: 1.14 to 1.27), clinical cure rate (*n* = 345, RR = 1.37, 95% CI: 1.19 to 1.57), and cure time (*n* = 513, MD = − 0.77, 95% CI: -0.89 to − 0.64). However, the quality of evidence for this finding was low due to high risk of bias of the included studies.

**Conclusions:**

The present work supported paediatric massage in treating acute diarrhoea in children. More well-designed randomized controlled trials are still needed to further evaluate the efficacy of paediatric massage.

**Electronic supplementary material:**

The online version of this article (10.1186/s12906-018-2324-4) contains supplementary material, which is available to authorized users.

## Background

Acute diarrhoea is a common disease in children in developing countries, especially for those younger than five years old [1]. There are many causes for acute diarrhoea in children [2], such as viruses or bacterial infection, malabsorption, and inflammatory bowel disease. Delayed treatment may cause dehydration, electrolyte imbalance, or even death. Currently, the main treatment for acute diarrhoea is pharmacotherapy, such as oral rehydration salts, Zinc supplement [3], probiotics, or loperamide [4].

In addition to pharmacotherapy, in China, massage therapy is also used by many traditional Chinese medicine (TCM) physicians to treat acute diarrhoea in children. Massage therapy is defined as the manipulation of the soft tissue of the body, and it is part of the complementary and alternative medicine. Most commonly, massage therapy is conducted on meridians and acupuncture points. The theory behind this therapy was outlined in Huangdi Neijing, which is an ancient Chinese medical book. Over the centuries, massage therapy has been used for emotional and physical healing [5, 6]. There are many benefits of massage therapy [7–10], such as enhancing immune function, unblocking meridians and collateral, activating Qi and blood, and improving the flow of Qi through the meridians. As a result, self-healing in the body is promoted. Paediatric massage is the massage therapy for children that aims to promote health [8, 11].

Many studies have assessed the effects of massage therapy. Vickers et al. [12] conducted a meta-analysis to assess the effects of massage therapy for improving health and development in preterm birth and low birth weight infants. The results showed that infants who received massage therapy demonstrated improved weight gain (5 g/d) and shorter hospital stays (4–5 days) compared to control groups who did not receive daily massage. Beider et al. [13] conducted a review to examine the effectiveness of massage therapy, and it was shown that massage therapy has real value to the paediatric population, such as a considerable impact on the state of anxiety. Moyer et al. [5] conducted a meta-analysis of randomized studies to test the effectiveness of massage therapy, and the results showed that massage therapy was superior in reducing anxiety and depression. Furthermore, several studies [14–16] have suggested that massage therapy is beneficial to children because it improves blood flow, normalizes function of the central nervous system, and reduces tissue stiffness.

For the treatment of acute diarrhoea in children, many clinical studies have reported beneficial effects of massage therapy. The theorized mechanism of massage therapy on acute diarrhoea is that it promotes gastrointestinal motility, regulates gastric acid secretion, and improves spontaneous bowel movements by stimulating acupuncture points [17–19], although the actual mechanism is still unclear. Considering no relevant systematic reviews have assessed the clinical effectiveness or the risk of paediatric massage therapy, in this study, a meta-analysis was conducted to assess the efficacy of paediatric massage for the treatment of acute diarrhoea in children.

## Methods

The protocol of this study was registered in PROSPERO with a registration number CRD42017056523.

### Database and search strategies

Relevant studies were searched in the following electronic databases: Cochrane Library, Web of Science, PubMed, Chinese Biomedical Literature Database, Chinese National Knowledge Infrastructure, Chinese Scientific Journal Database, and Wan-fang Database up to April 30, 2018. The following search terms were used: (massage OR tui na OR manipulation OR acupressure) AND (infant OR child OR baby OR paediatrics) AND (diarrhea OR diarrhoea) AND (randomized controlled trial). There were no language limitations.

### Inclusion criteria

Included studies must all be randomized controlled trials (RCTs).

#### Participants

Paediatric patients who were diagnosed with acute diarrhoea. Acute diarrhoea is a disease defined as more stools than normal, and there are some changes in the stool traits, such as loose stool, or watery stool. Usually, course of the disease lasts less than 14 days.

#### Interventions

Interventions using massage therapy alone or combined with other non-pharmacological approaches were included. Interventions using massage therapy combined with pharmacological therapies, such as montmorillonite, were excluded.

#### Comparators

In the control groups, patients received pharmacotherapy.

#### Outcomes

Clinical effective rate was the primary outcome. Some other outcomes included clinical cure rate, and cure time.

### Exclusion criteria

Patients with chronic diarrhoea were excluded. Studies lacking data for course of disease were excluded, because it is impossible to judge patients were in acute diarrhoea. Non-RCTs, unpublished or repeated literature, case studies, qualitative studies, and experience summaries were excluded.

### Data extraction and quality assessment

Three reviewers (Gao, Jia, and Huang) independently extracted the data and conducted quality assessments. Statistical analysis was conducted using the RevMan 5.3 software, and risk of bias was assessed using the Cochrane tool. Any disagreement was resolved by discussions among the reviewers.

## Results

### Description of included studies

**Fig. 1:**
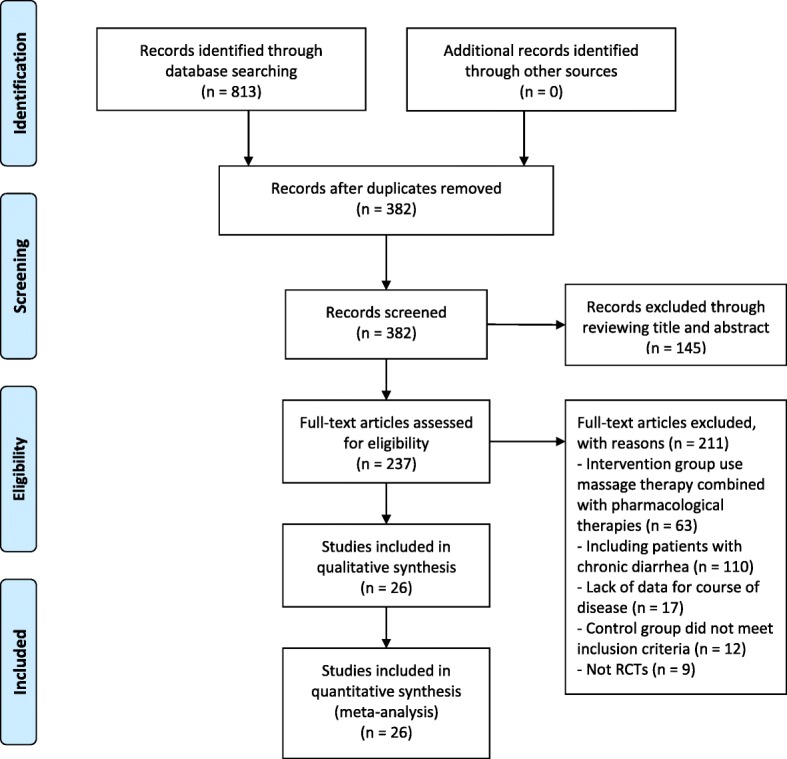
PRISMA flow diagram

In this meta-analysis, 813 potentially eligible studies were identified, of which 787 were excluded, including 431 repeated publications, 145 irrelevant studies, 63 studies that combined pharmacological therapy in the intervention group, 110 studies that included patients with chronic diarrhoea, 17 studies that lacked data for the course of disease, 12 studies that did not meet inclusion criteria in the control group, and 9 studies that were not RCTs. Thus, a total of 26 studies [20–45] encompassing 2644 participants (1462 in the intervention group and 1182 in the control group) were included in the meta-analysis, and all were published in Chinese Journal Literature Databases. The screening process is summarized in a flow diagram (Fig. 1).

Details of the 26 studies are summarized in Table 1. All the children were under 5 years old, and disease course of the participants was less than 14 days. In the intervention group, massage therapy was used alone or combining with other non-pharmacological approaches to treat acute diarrhoea. There are many different treatment methods in the intervention group, and details of the interventions of the included studies are shown in additional file 1. In general, these interventions can be classified into several categories. According to the book Massage [46] in China, there were some basic manipulations suggested for the treatment of acute diarrhoea, including pushing Pijing, Dachang upward, rubbing the abdomen, kneading navel, pushing Qijiegu upward, and Pinching spine. Almost all the studies used the basic massage treatment. Furthermore, in the aim of increasing efficacy, many physicians utilized acupressure (Pressing some acupuncture points), while some physicians performed an individual massage treatment, some other physicians used the acupuncture therapy. In the control group, all the studies used pharmacotherapy; fourteen studies [21, 23, 25, 26, 28–30, 34, 36–38, 42–44] used montmorillonite alone, while other studies used combined therapy.

**Table 1:** Details of the 26 included studies

Study	Sample size	Age	Course of disease	Intervention group	General classification of the massage therapy	Control group
Cheng (2014)	72 (36/36)	1.9 ± 0.5 y	3.6 ± 1.1 d	massage 3 d	BMT + PSAP	(1) montmorillonite (2) combined bacillus subtilis and enterococcus faecium 3 d
Du (2009)	100 (52/48)	19 ± 5 m	1.2 ± 0.5 d	massage 3 d	BMT + PSAP	montmorillonite 3 d
Gao (2005)	60 (30/30)	18 ± 6 m 17 ± 6.5 m	1.3 ± 0.5 d 1.2 ± 0.6 d	massage 3 d	BMT + PSAP	(1) montmorillonite (2) combined bacillus subtilis and enterococcus faecium 3 d
Leng (2011)	26 (20/6)	3 m to 5 y	≤1w	massage 3 d	BMT + PSAP	montmorillonite 3 d
Li K (2013)	61 (30/31)	< 3 y	< 2 w	massage 3 d	BMT + PSAP	(1) Cangling antidiarrhea oral solution (2) montmorillonite (3) bifidobacterium 3 d
Li X (2015)	176 (87/89)	2 m to 3 y 1.5 m to 2 y and 11 m	< 2 w	massage 5 d	IMT	montmorillonite 5 d
Ma (2016)	80 (40/40)	3 m to 4 y	< 1 w	massage 3 d	BMT + PSAP	montmorillonite 3 d
Ni (2018)	70 (35/35)	1.67 ± 1.41 y 1.64 ± 1.39 y	3.16 ± 0.82 d 3.19 ± 0.87 d	massage 7 d	BMT + PSAP	norfloxacin 7d
Peng (2011)	240 (180/60)	3 m to 5 y	2.81 ± 1.61 d 2.78 ± 1.39 d	massage 3 d	BMT + PSAP	montmorillonite 3 d
Shao (2006)	120 (68/52)	18 ± 6 m 17 ± 6 m	1.3 ± 0.5 d 1.2 ± 0.6 d	massage 3 d	BMT + PSAP	montmorillonite 3 d
Tang (2014)	135 (67/68)	1.84 ± 0.33 y 1.87 ± 0.37 y	6.84 ± 2.21 d 6.58 ± 2.13 d	massage	BMT + PSAP	montmorillonite
Tao (2015)	60 (30/30)	2.03 ± 1.21 y 2.30 ± 1.16 y	2.81 ± 1.31 d 2.70 ± 1.20 d	massage 3 d	BMT + PSAP	(1) montmorillonite (2) clostridium butyricum 3 d
Wang (2004)	88 (48/40)	6 m to 3 y	all were acute diarrhea	massage 3 d	BMT + PSAP	(1) intravenous drip ribavirin (2) montmorillonite (3) lactobacillus 3 d
Wang (2014)	86 (43/43)	1.75 ± 0.37 y 1.25 ± 0.14 y	3.47 ± 0.34 d 3.41 ± 0.24 d	massage 3 d	BMT + PSAP	(1) norfloxacin (2) ciprofloxacin 5 d
Yang (2016)	80 (40/40)	1.60 ± 0.72 y 1.81 ± 0.68 y	< 1 w	massage 3 d	BMT + PSAP	montmorillonite 3 d
Yang (2013)	69 (36/33)	6 m to 3 y 6 m to 3.5 y	1–5 d 1–6 d	massage 6 d	BMT + PSAP	(1) montmorillonite (2) combined bacillus subtilis and enterococcus faecium (3) bifidobacterium 6 d
Yin (2000)	50 (30/20)	< 3 y	< 3 d	massage 3 d	BMT + PSAP	montmorillonite 3 d
Yin (2009)	315 (190/125)	2 m to 5 y	< 48 h	massage 3 d	IMT	montmorillonite 3 d
You (2013)	55 (32/23)	11.55 ± 4.68 m 10.58 ± 5.05 m	3.25 ± 1.06 d 3.39 ± 1.15 d	massage 3 d	BMT + PSAP	montmorillonite 3 d
Zhang (2016)	180 (90/90)	2.41 ± 1.6 y 2.5 ± 1.2 y	2.8 ± 1.4 d 2.9 ± 1.2 d	massage 6 d	BMT + PSAP	(1) montmorillonite (2) bifidobacterium 6 d
Zhang (2011)	64 (32/32)	< 3 y	1 to 3 d	massage 3 d	BMT + PSAP	(1) probiotics (2) mucosal protection 3 d
Zhao (2016)	80 (40/40)	3.1 ± 0.6 y 3.3 ± 0.7 y	14.2 ± 2.1 h 13.2 ± 1.7 h	massage 3 d	BMT + PSAP	(1) enterococcus faecium (2) bifidobacterium (3) montmorillonite 3 d
Zhu (2004)	90 (60/30)	< 3 y	≤3 d	massage 3 d	BMT + PSAP	montmorillonite 3 d
Li G (2013)	120 (60/60)	1.5 ± 0.3 y	< 3 d	massage + acupuncture 3 d	IMT + Acupuncture	montmorillonite 3 d
Wang (2003)	99 (52/47)	1.7 ± 1.1 y 1.8 ± 1.2 y	< 3 d	massage + acupuncture 3 d	BMT + IMT + Acupuncture	montmorillonite 3 d
Wei (2016)	68 (34/34)	12.6 ± 5.7 m 11.9 ± 5.3 m	3.1 ± 0.3 d 3.2 ± 0.4 d	massage + acupuncture 7 d	BMT + IMT + Acupuncture	combined bacillus subtilis and enterococcus faecium 7 d

*y*: years; m: months; *w* weeks; *d* days; *h* hours; *BMT* Basic massage treatment; *PSAP* Pressing some acupuncture points; *IMT* Individual massage treatment

Ten studies [21, 24, 26, 30, 34–36, 38, 40, 42] reported using standard of Diagnosis and treatment proposal for diarrhoea in China [47] for the definition of acute diarrhoea. This standard provides a diagnostic basis for acute diarrhoea, that is more stools than usual, some changes in the stool traits, and lasting less than 2 weeks. Seven studies [25, 28, 29, 33, 37, 43, 44] adopt a standard in traditional Chinese medicine [48], while other studies did not report any standards cited in the inclusion criteria. However, their definitions of acute diarrhoea in the inclusion criteria were similar to the standard of Diagnosis and treatment proposal for diarrhoea in China.

### Risk of bias

**Fig. 2:**
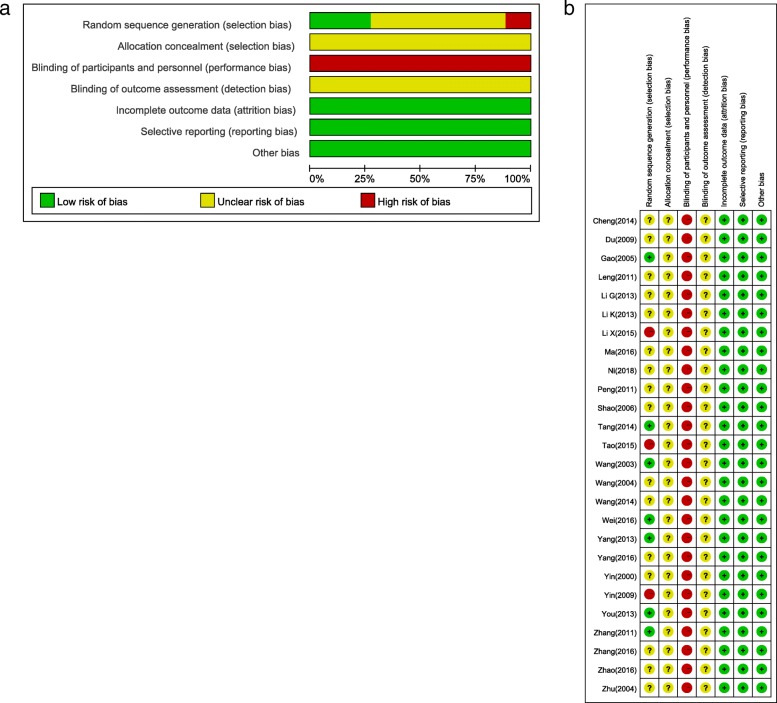
Risk of bias graph: (**a**) risk of bias of all included studies; (**b**) Risk of bias summary

The risk of bias was high in the included studies (Fig. 2). All the studies used randomization, but only seven [22, 30, 35, 38, 40, 44, 45] of these studies reported using an appropriate method of random sequence generation, while three [25, 31, 36] of these studies reported using inappropriate methods. None of the studies described the method for allocation concealment and blinding of the outcome assessment. Most of the included studies had a high risk of performance bias, because both the physicians and the patients clearly knew which treatment was given.

### Outcome measurements

#### Clinical effective rate

According to the standard of Diagnosis and treatment proposal for diarrhoea in China [47], effective was defined as that there is significant improvement in stool traits after 72 h of treatment, and the frequency of stools reduced by 50%. Only four of all studies did not use this standard to define effective. They adopt a similar method, the difference is that two studies [35, 39] did the evaluation after 6 days of treatment, while two studies [27, 45] did the evaluation after 7 days of treatment.

**Fig. 3:**
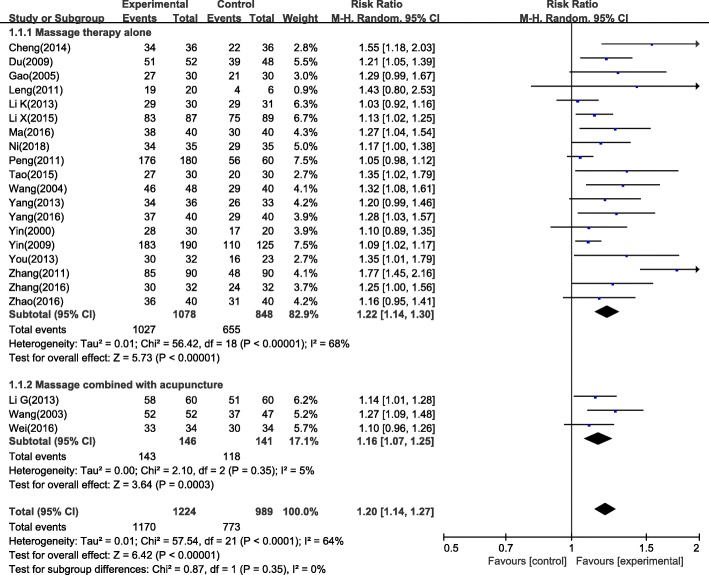
Forest plot of clinical effective rate

Twenty-two studies showed that massage therapy had a higher clinical effective rate compared with pharmacotherapy. Since high heterogeneity was observed in the meta-analysis (I^2^ = 64%, which is higher than 50%), a random-effects model was used to calculate the pooled estimation with analysis of dichotomous data using relative risk (RR), including 95% confidence intervals (CIs). The meta-analysis showed favourable effects of massage therapy in clinical effective rate (*n* = 2213, RR = 1.20, 95% CI: 1.14 to 1.27, *P* < 0.01) compared with the control group (Fig. 3). A subgroup analysis was conducted for massage therapy alone and massage combined with acupuncture. The results showed high heterogeneity in the subgroup massage therapy alone, with I^2^ = 68%. While there was low heterogeneity for subgroup differences, with I^2^ = 0%.

**Fig. 4:**
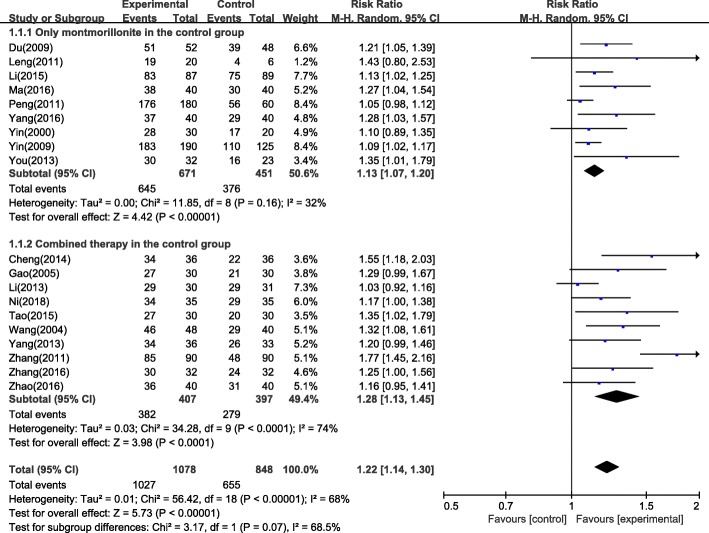
Subgroup analysis for montmorillonite alone and combined therapy

The reason for the high heterogeneity in the subgroup massage therapy alone may be due to different pharmacotherapies in the control group. Since several studies used montmorillonite alone in the control group, a subgroup analysis was also performed for montmorillonite alone and combined therapy, as shown in Fig. 4. The subgroup of montmorillonite had 1122 patients, with RR = 1.13, 95% CI: 1.07 to 1.20, *P* < 0.01. The subgroup with combined therapy had 804 patients, with RR = 1.28, 95% CI: 1.13 to 1.45, P < 0.01. Heterogeneity in the subgroup montmorillonite was low (I^2^ = 32%), while heterogeneity in the subgroup of combined therapy was high (I^2^ = 74%), which resulted in a significant difference between these two subgroups, with I^2^ = 68.5%. Thus, it can be concluded that the differences in the pharmacotherapies in the control group was a main reason for the high heterogeneity.

#### Clinical cure rate

**Fig. 5:**
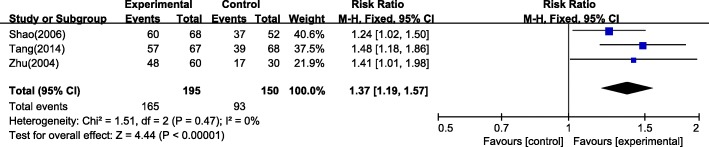
Meta-analysis of clinical cure rate

Cure was defined as that the stool traits and frequency returned to normal within 72 h of treatment. Three studies [29, 30, 42] showed that massage therapy had a higher clinical cure rate compared with pharmacotherapy (*n* = 345, RR = 1.37, 95% CI: 1.19 to 1.57, I^2^ = 0%), as shown in Fig. 5.

#### Cure time

**Fig. 6:**
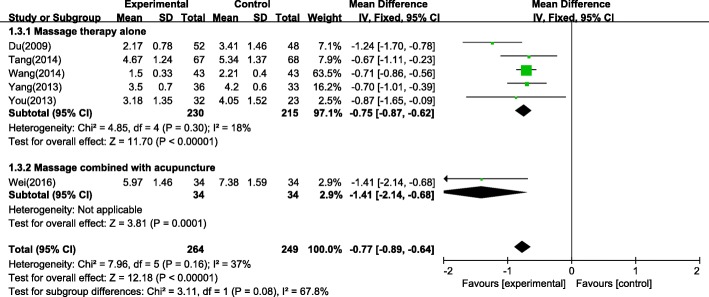
Forest plot of cure time (days)

Cure time was defined as the length of treatment time spent before the patient is completely cured. Six studies [21, 30, 33, 35, 38, 45] reported data on cure time. The meta-analysis of cure time shows a mean difference (MD) of − 0.77 (95% CI: -0.89 to − 0.64) with a low heterogeneity (I^2^ = 37%), as shown in Fig. 6, which means that the participants receiving massage therapy had shorter cure time than those with pharmacotherapy.

## Discussion

In TCM, paediatric massage therapy takes into consideration each child’s individual physical development and has been found to have many benefits through manipulation on acupoints, such as improving digestive system [49], promoting mental and physical health [11], and increasing body weight in premature infants [50]. However, massage therapy should be carried out by medical staff with enough training, for inaccurate treatment may reduce the clinical effects. As a complementary and alternative medicine, massage therapy benefits the body in a way that is quick, easy, and inexpensive. It was reported that paediatric massage therapy has been used for treating diseases in children for thousands of years [51]. In recent years, many physicians have reported that paediatric massage therapy can effectively improve the function of the spleen and stomach by stimulating some specific acupoints [19, 49]. However, clinical effectiveness and risk have not been systematically assessed. This study reported a meta-analysis of massage therapy for the treatment of acute diarrhoea in children.

In the treatment of acute diarrhoea in children, massage therapy acts on the on meridians and acupuncture points, activating the flow of Qi and nourishing spleen and stomach. As a result, the function of the digestive system is improved. In addition, several studies have reported that massage therapy has a positive effect on emotional state of children [52, 53], and as we know, a good emotional state has benefits for improving immune function. It is reasonable to think that part of the rationale for massage therapy to address acute diarrhoea is improving emotional state in paediatric patients.

High heterogeneity was found in clinical effective rate, with I^2^ = 64%. Reasons may include different pharmacotherapies used in different control groups. For example, Du [21] used montmorillonite in the control group for the treatment of diarrhoea, while Zhang [40] used probiotics and mucosal protection. These different therapies make the efficacy of massage therapy hard to assess. We conducted a subgroup analysis for montmorillonite alone and combined therapy. It was shown that heterogeneity in the subgroup montmorillonite was low (I^2^ = 32%), while heterogeneity in the subgroup combined therapy was high (I^2^ = 74%), resulting in a significant difference between these two subgroups. It can be concluded that the differences in pharmacotherapies in the control group was a main reason for the high heterogeneity. Second,

In addition, another reason for high heterogeneity was that different massage techniques were used by different TCM physicians (as shown in additional file 1). In these studies, some basic manipulations were utilized by all the physicians. However, more manipulations were conducted in different ways, massaged parts of body, manipulation order, and manipulation frequency are usually different. Furthermore, diarrhoea is classified into different types by different TCM physicians, such as cold damp type, spleen deficiency type, and damp hot type; thus, different massage therapy techniques were applied to different types of diarrhoea.

The methodological quality for this finding was low because of high risk of bias. There are several limitations in this systematic review. First, for most of the included studies, the methods for randomization, allocation concealment, and blinding were not reported clearly. Due to the characteristics of TCM, both the physicians and the patients clearly knew which treatment was been given, making blinding methods difficult. Second, in the 26 included studies, only 8 studies had sample sizes greater than 100 trials; small sample sizes in most studies made it hard to draw a meaningful conclusion. Third, clinical effective rate was the main outcome measurement for most studies, and thus, bias from the physicians might decrease reliability and validity of the studies. Fourth, limited information about adverse effects was reported by the included studies; therefore, conclusions on the safety of massage therapy on treatment of acute diarrhoea should be seriously considered. Fifth, all the studies were conducted in China, which may limit generalization of the findings. Considering the limitations in this meta-analysis, it is strongly recommended that more rigorous RCTs with large sample sizes should be used to further evaluate the clinical efficacy and adverse effects of paediatric massage in treating acute diarrhoea in children.

## Conclusions

A total of 26 studies encompassing 2644 patients were included in this meta-analysis that compared paediatric massage and pharmacotherapy for treating acute diarrhoea in children. The results of the meta-analysis suggest that massage therapy was superior to pharmacotherapy. However, the studies analysed to date are of relatively low quality. More rigorous RCTs with large sample sizes are recommended to further evaluate the clinical efficacy and adverse effects of paediatric massage in treating acute diarrhoea in children.

## Additional file

Additional file 1: Details of the massage therapy. Details of the interventions of the included studies. (DOCX 19 kb)

## Abbreviations

RCTs: Randomized controlled trials
TCM: Traditional Chinese medicine
