# Supplementary material for: Paediatric massage for treatment of acute diarrhoea in children: a meta-analysis
Source: BMC Complement Altern Med. 2018 Sep 18;18:257. doi: 10.1186/s12906-018-2324-4 (PMC6145333; doi:10.1186/s12906-018-2324-4)
Supplement: Supplementary file 1 — Details of the massage therapy. Details of the interventions of the included studies. (DOCX 19 kb) [file 12906_2018_2324_MOESM1_ESM.docx]

Additional file 1 Details of the massage therapy

| Study | Details of the massage therapy |
| --- | --- |
| Cheng  (2014) | Push Pijing and Weijing upward 300 times respectively, Arc-pushing inner gossip 150 times, Push Xiaochang and Dachang downward 100 times respectively, Knead Zusanli (ST36) 100 times, Knead Tianshu (ST25) and Shenque (RN08) 50 times respectively, Rub abdomen counterclockwise 300 times, Push Qijiegu upward 200 times, Press Guiwei 200 times, Pinch spine 5 times. |
| Du  (2009) | Push Pijing and Dachang upward 100 to 500 times respectively, Push Banmen and Sanguan 100 to 300 times respectively, Rub abdomen counterclockwise 300 to 500 times, Knead navel counterclockwise 100 to 300 times, Knead Guiwei 100 to 300 times, Push Qijiegu upward 100 to 300 times, Pinch spine 3 to 7 times. |
| Gao  (2005) | Spiral push Pijing, Push Sanguan, Push Dachang, Knead Wailaogong (EX-UE08), Knead navel, Rub abdomen, Push Qijiegu upward, Knead Guiwei, each manipulation lasts more than 3 minutes, Knead Zusanli (ST36) 50 times. |
| Leng  (2011) | Push Pijing, Dachang, and Sanguan upward respectively, Knead Lanmen and navel respectively, Rub abdomen, Push Qijiegu upward, Knead Guiwei and Zusanli (ST36) respectively, each manipulation lasts 100 to 200 times. |
| Li K  (2013) | Push Pijing downward and upward 100 times respectively, Push Dachang downward and upward 200 times respectively, Knead Banmen 100 times, Arc-pushing inner gossip 200 times, Push Liufu downward 200 times, Rub abdomen 200 times, Pinch spine 6 times, Push Qijiegu upward 100 times, Knead Guiwei 100 times. |
| Li X  (2015) | Cold damp type: Push Pijing and Weijing upward respectively, Push Sanguan, Push Dachang upward, Knead navel, Push Qijiegu upward, Knead Guiwei, Zusanli (ST36), and Wailaogong (EX-UE08). Damp hot type: Push Liufu downward, Push Pijing, Weijing, Dachang, and Xiaochang downward respectively, Knead Tianshu (ST25), Push Tianheshui. Improper diet type: Push Pijing upward, Push Dachang downward, Knead Banmen, Arc-pushing inner gossip, Knead Zhongwan (RN12), Press Tianshu (ST25), Knead Guiwei, Rub abdomen. The total massage therapy lasts 20 minutes. |
| Ma  (2016) | Rub abdomen clockwise 3 minutes, Rub abdomen counterclockwise 2 minutes, Push Qijiegu clockwise 50 times, Knead Guiwei counterclockwise 30 times, Push governor meridian clockwise 5 times, Pinch spine 5 times, Push governor meridian counterclockwise 3 times, Push Sanguan clockwise 200 times, Push Liufu counterclockwise 200 times. |
| Ni  (2018) | Push Weijing, Dachang, Pjing 300 times respectively, Rub abdomen 5 minutes, Pinch spine. |
| Peng  (2011) | Push Pijing and Dachang upward respectively, Knead Lanmen, Knead navel, Rub abdomen, Push Qijiegu upward, Knead Guiwei, Pinch spine, Knead Zusanli (ST36). |
| Shao  (2006) | Push Tianmen, Kangong, and Taiyang (EX-HN05) 20 to 30 times respectively, Press Jianjing (GB21) 3 to 5 times, Push Pijing upward 40 times, Push Ganjing downward 250 times, Push Xinjing downward 100 times, Push Feijing upward 200 times, Push Shenjing upward 300 times, Push Dachang 150 times, Knead Zusanli (ST36) 60 times, Knead Zhongtuo 200 times, Knead navel 200 times, Knead Guiwei 100 times, Push Qijiegu upward 50 times. |
| Tang  (2014) | Push Pijing and Dachang upward 300 times respectively, Push Banmen 300 times, Rub abdomen clockwise 3 minutes, Knead Zusanli (ST36) clockwise 3 minutes, Knead Baihui (DU20) clockwise 3 minutes, Knead Shenshu (BL23) clockwise 3 minutes, Knead Guiwei 300 times, Pinch spine 3 times. |
| Tao  (2015) | Push Dachang downward 100 times, Rub abdomen 500 times, Push Qijiegu upward 300 times, Knead Banmen 100 times, Push Pijing upward 300 times, Knead Zusanli (ST36) 200 times, Pinch spine 6 times. |
| Wang  (2004) | Push Pijing and Dachang upward respectively, Push Banmen, Arc-pushing inner gossip, Push Qijiegu upward, Knead Guiwei, Rub abdomen, Knead navel, each manipulation lasts 100 to 200 times. |
| Wang  (2014) | Push Pijing and Dachang upward 300 times respectively, Rub abdomen and knead navel 5 minutes respectively, Knead Guiwei 100 times, Push Qijiegu upward 100 times. |
| Yang  (2016) | Rub abdomen clockwise 3 minutes, Rub abdomen counterclockwise 2 minutes, Push Qijiegu upward 50 times, Knead Guiwei 30 times, Pinch spine upward 5 times, Pinch spine downward 3 times, Push Sanguan upward 200 times, Push Liufu downward 200 times. |
| Yang  (2013) | Push Pijing and Dachang upward 300 times respectively, Rub abdomen counterclockwise 2 minutes, Push Qijiegu upward 200 times, Digital press Guiwei 9 times. |
| Yin  (2000) | Rub abdomen clockwise 3 minutes, Knead Guiwei 1 minute, Push spine 10 times, Pinch spine 3 times. |
| Yin  (2009) | Damp hot type: Push Pijing downward 250 times, Push Ganjing and Xinjing downward 200 times respectively, Push Feijing downward 100 times, Push Shenjing upward 150 times, Push Dachang downward 200 times, Push Liufu 120 times, Knead Zusanli (ST36) and Zhongwan (RN12) 120 times respectively, Knead navel 200 times, Push Houxi (SI03) downward 150 times, Grasp Dujiao 3 to 5 times, Knead Guiwei 100 times, Press Jianjing (GB21) 2 to 3 times.  Cold damp type: Push Pijing upward 300 times, Push Ganjing downward 250 times, Push Feijing upward 150 times, Push Shenjing upward 200 times, Push Dachang 150 times, Rub abdomen 2 minutes, Knead Zusanli (ST36) 60 times, Knead Zhongwan (RN12) 150 times, Knead navel 200 times, Knead Guiwei 100 times, Push Qijiegu upward 50 times, Push Feishu (BL13) until redness, Press Jianjing (GB21) 2 to 3 times. Spleen deficiency type: Push Pijing upward 400 times, Push Feijing upward 200 times, Push Shenjing upward 350 times, Push Dachang 150 times, Knead Wailaogong (EX-UE08) 100 times, Knead Zhongwan (RN12) 300 times, Rub abdomen 100 times, Pinch spine 5 times, Knead navel 200 times, Knead Guiwei 120 times, Push Qijiegu upward 50 times, Push Feishu (BL13) until redness, Press Jianjing (GB21) 2 to 3 times. Combining vomiting type: Push Pijing downward 300 times, Push Xinjing downward 250 times, Push Feijing downward 350 times, Push Shenjing upward 200 times, Push Dachang upward 200 times, Push Houxi (SI03) downward 150 times, Push Liufu 90 times, Knead Zusanli (ST36) 80 times, Knead Guiwei 100 times, Knead Banmen 100 times, Push Feishu (BL13) until redness, Press Jianjing (GB21) 2 to 3 times. |
| You  (2013) | Rub abdomen 5 minutes, Knead navel 200 times, Push Pijing upward 200 times, Knead Zusanli (ST36) 200 times, Knead Guiwei 200 times, Push Qijiegu 100 times, Pinch spine 5 times. |
| Zhang  (2016) | Push Weijing downward and upward 300 times respectively, Push Pijing upward 300 times, Arc-pushing Banmen 200 times, Arc-pushing inner gossip 200 times. |
| Zhang  (2011) | Seperate Yinyang 300 times, Push Dachang downward 300 times, Push Pijing downward and upward 500 to 1000 times respectively, Move earth into water 100 to 300 times, Rub abdomen 300 times, Push Qijiegu upward 300 times, Pinch spine 3 to 5 times. |
| Zhao  (2016) | Massage navel and abdomen 5 minutes respectively, Knead the part between Qijiegu and tail vertebrae 200 times, Knead Guiwei 100 times, Pinch spine 6 times. |
| Zhu  (2004) | Digital press Zhixie, Zusanli (ST36), and Sanyinjiao (SP06) 2 to 3 minutes respectively, Digital press Dantian with middle finger 1 to 2 minutes, Pinch spine 3 to 5 times, Seperate Yinyang 100 to 200 times, Push Dachang downward 200 to 300 times, Push Xiaochang downward 100 to 200 times, Push Pijing upward 400 to 500 times. |
| Li G  (2013) | Seperate Yinyang 30 to 50 times;  Acupuncture Zusanli (ST36), Sanyinjiao (SP06), and Zhixiexue. |
| Wang  (2003) | Pinch spine 3 to 5 times, Seperate Yinyang 100 to 200 times, Push Dachang downward 200 to 300 times, Push Xiaochang downward 100 to 150 times, Move earth into water 100 to 200 times, Push Pijing upward 400 to 500 times;  Acupuncture Zusanli (ST36), Sanyinjiao (SP06), and Zhixiexue. |
| Wei  (2016) | Push Pijing and Dachang upward, Push Xiaochang downward, Knead Banmen, Move earth into water, Arc-pushing inner gossip, Push Sanguan, Push Qijiegu, Pinch spine;  Acupuncture Tianshu (ST25), Zusanli (ST36), Shangjuxu (ST37), Sanyinjiao (SP06), Qihai (RN6), Zhongwan (RN12), and Zhixiexue. |

Upward: To the direction of the heart; Downward: To the opposite direction of the heart.
